# Case Report: Diffuse peribiliary metastasis from ovarian cancer mimicking sclerosing cholangitis without a detectable mass

**DOI:** 10.3389/fonc.2026.1901172

**Published:** 2026-10-05

**Authors:** Xiao Li, Zhijie Zhang

**Affiliations:** Department of Gastroenterology, Liuzhou Hospital of Traditional Chinese Medicine, Liuzhou, Guangxi, China

**Keywords:** bile duct metastasis, case report, glissonian sheath, obstructive jaundice, ovarian cancer

## Abstract

**Background:**

Ovarian cancer typically spreads via peritoneal seeding, hematogenous, or lymphatic routes. Bile duct metastasis is extremely rare, and peribiliary metastasis manifesting as diffuse Glissonian sheath infiltration without a detectable mass lesion, to our knowledge, has not been well documented.

**Case presentation:**

A 42-year-old woman with stage IVB high-grade serous ovarian carcinoma, previously treated with cytoreductive surgery and multiple lines of chemotherapy, presented with fatigue, anorexia, and mild jaundice. Magnetic resonance cholangiopancreatography (MRCP) revealed bile duct wall thickening, peribiliary edema (Glissonian sheath edema), and diffuse inflammatory changes without a discrete mass. Autoimmune and infectious workups were negative. Endoscopic retrograde cholangiopancreatography (ERCP) with biopsy showed micropapillary clusters of atypical cells. Immunohistochemistry was positive for CK7, CK19, PAX-8, P16, and mutant-type p53; CEA focally positive; ER negative; Ki-67 high. This confirmed metastatic high-grade serous carcinoma of ovarian origin involving the common bile duct wall. Imaging (MRI/MRCP) suggested diffuse peribiliary involvement compatible with Glissonian sheath infiltration, although the small biopsy did not directly sample the intrahepatic sheath itself. We hypothesize that the radiological changes reflect peribiliary lymphatic spread, but this remains an inference based on imaging-pathology correlation rather than a histologically proven mechanism. Effective biliary drainage could not be achieved because the patient was unable to tolerate the nasobiliary drain. She was discharged against medical advice and died shortly thereafter, according to family report.

**Conclusions:**

In cancer patients with unexplained cholestasis, peribiliary metastasis should be considered even when no mass is seen on cross-sectional imaging. ERCP with biopsy and immunohistochemistry are essential for diagnosis.

## 1 Introduction

Ovarian cancer is the most lethal gynecologic malignancy, with advanced-stage disease often spreading via peritoneal seeding, hematogenous dissemination, and lymphatic routes (1). Common metastatic sites include the peritoneum, omentum, liver parenchyma, and lungs. Autopsy studies have shown that the peritoneum is involved in up to 93.9% of cases (2), the omentum in 73.6% (2), the liver in 25.4%–73% (2, 3), and the lungs in up to 44% of cases (3). Bile duct involvement, however, is exceptionally rare. To date, only a handful of cases have described ovarian cancer metastasis to the biliary tree, most of which presented as discrete hilar masses mimicking cholangiocarcinoma (4, 5).

Shijo et al. reported a 55-year-old woman with ovarian serous adenocarcinoma who developed a 10-mm solid tumor at the hepatic hilum causing obstructive jaundice; the mass was confirmed histologically as metastatic ovarian cancer following surgical resection (4). Puvvada et al. described a 63-year-old patient in whom metastatic ovarian cancer to the caudate lobe led to external compression of the common bile duct and duodenum, managed with ERCP and biliary stenting (5). In both reports, the metastatic lesions presented as identifiable mass lesions—either a discrete hilar tumor or a parenchymal deposit causing extrinsic compression. While mass-forming and infiltrative patterns are not necessarily mutually exclusive, the clinically relevant biliary component in our case did not present as a discrete mass detectable by conventional imaging.

The present case describes a distinctly different pattern: diffuse peribiliary tumor infiltration involving the Glissonian sheath, without any detectable mass on cross-sectional imaging, radiologically mimicking sclerosing cholangitis. We searched PubMed and Web of Science using combinations of the following keywords: ovarian cancer, high-grade serous carcinoma, bile duct metastasis, biliary metastasis, peribiliary metastasis, Glissonian sheath, periportal infiltration, obstructive jaundice, and sclerosing cholangitis. To our knowledge, this pattern of “occult” biliary metastasis has not been well characterized in the literature. We report this case to alert clinicians to this important consideration in cancer patients presenting with unexplained cholestasis.

## 2 Case description

### 2.1 Patient information and past medical history

A 42-year-old Chinese woman who had been diagnosed with high-grade serous ovarian carcinoma at another hospital in April 2022 presented with one week of worsening fatigue, anorexia, aversion to greasy foods, and intermittent right upper quadrant pain. She was diagnosed in April 2022 at a tertiary hospital and underwent cytoreductive surgery, including laparoscopic omental and intra-abdominal tumor resection. She received a first cycle of chemotherapy with bevacizumab, paclitaxel, and carboplatin on May 8, 2022, followed by a second cycle with paclitaxel and carboplatin on May 30, 2022. In June 2022, she underwent additional resections: laparoscopic total splenectomy, liver tumor resection, diaphragmatic lesion resection, and enterolysis. Pathology from these specimens showed no viable tumor cells, with only degeneration, necrosis, and fibrosis, indicating a pathologic complete response (pCR) to neoadjuvant chemotherapy. Germline BRCA1/2 testing at that time revealed a pathogenic BRCA1 mutation. She then completed four additional cycles of paclitaxel and carboplatin from July 25 to September 30, 2022. Approximately 6–8 weeks after completing chemotherapy, she started maintenance therapy with olaparib. In March 2024, disease progression was documented with new retroperitoneal lymphadenopathy and bone metastases. She then received chemotherapy with bevacizumab, paclitaxel, and carboplatin. Imaging in June 2024 showed a partial response, but the patient declined further chemotherapy and subsequently started niraparib, which she had obtained on her own. In September 2025, magnetic resonance imaging revealed new metastases in the mesentery, peritoneum, and mediastinal lymph nodes. She then received three cycles of albumin-bound paclitaxel (400 mg), carboplatin (500 mg), and bevacizumab (500 mg) on September 4, September 27, and October 24, 2025. She was discharged after completing these three cycles of chemotherapy in late October 2025. The patient’s platinum sensitivity status at the time of final treatment was not clearly documented in the available medical records. The stage IVB (T3bN0M1b) designation was documented in the discharge summary from her final oncology admission in October 2025, based on the cumulative metastatic burden at that time (retroperitoneal lymph nodes, bone, mesenteric, peritoneal, and mediastinal metastases). The initial staging from April 2022 was not available in our records, as the patient was diagnosed at another hospital. She was readmitted to our gastroenterology department on November 24, 2025, due to worsening fatigue, anorexia, and jaundice. Laboratory workup on admission revealed significant cholestatic liver dysfunction.

### 2.2 Physical examination and laboratory findings

Upon admission, the patient’s vital signs were stable, with tachycardia (heart rate 119 beats per minute). Mild scleral icterus was observed. Laboratory findings indicated cholestatic liver injury (elevated ALP, GGT, and total bilirubin). Creatinine was 74 μmol/L, CRP 6.84 mg/L, and CA-125 3666.70 U/mL. Other blood tests, including cardiac biomarkers, electrolytes, and random blood glucose, were within normal limits. The temporal changes in liver function parameters during the hospital course are presented in **Supplementary Table 1**.

Serial CA-125 measurements were available from the patient’s previous admissions (**Supplementary Figure 1**). CA-125 remained within normal limits at 17.8 U/mL in January 2023 and 21.6 U/mL in July 2023, during olaparib maintenance therapy. In March 2024, CA-125 rose sharply to 283.5 U/mL, concurrent with radiographic evidence of disease progression (retroperitoneal lymphadenopathy and bone metastases). After chemotherapy, CA-125 decreased to 348.3 U/mL in April 2025, correlating with a partial radiographic response. However, CA-125 subsequently rose to 1000.0 U/mL in September 2025, coinciding with new metastases in the mesentery, peritoneum, and mediastinal lymph nodes. On admission to our department, CA-125 further increased to 3666.70 U/mL. This longitudinal trend demonstrates a consistent correlation between CA-125 levels and the patient’s clinical course, with elevation preceding or coinciding with radiographic progression. The markedly elevated CA-125 at admission reflected ongoing disease progression rather than a discordant or mixed response, consistent with the patient’s overall clinical deterioration.

### 2.3 Imaging findings

A contrast-enhanced CT of the chest, abdomen, and pelvis performed during the patient’s oncology admission approximately one month prior to her gastroenterology admission (October 2025) showed postoperative changes of ovarian cancer, with mesenteric, retroperitoneal, and mediastinal lymph node metastases that had decreased in size compared with previous imaging. Gallbladder stones, cholecystitis, and cholangitis were noted (**Figure 1a**). Importantly, the CT revealed mild thickening and enhancement of the intrahepatic and extrahepatic bile duct walls (**Figure 1b**). However, no discrete biliary mass or intrahepatic mass lesion was identified, and no bile duct dilation or portal vein stenosis was reported. In retrospect, these subtle bile duct wall changes were interpreted as inflammatory at the time.

**Figure 1:**
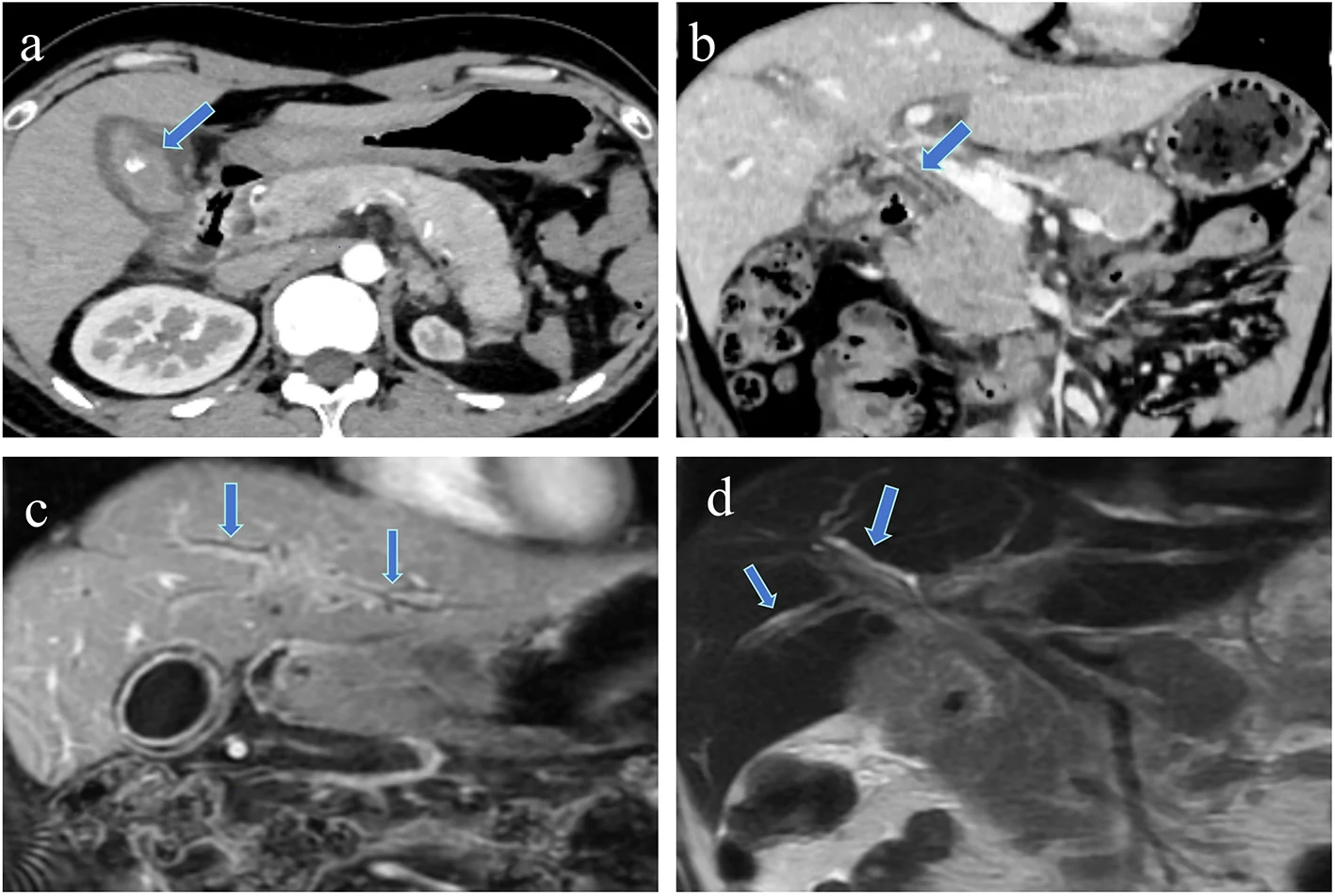
**(a)** Axial contrast-enhanced CT image (arterial phase) shows gallbladder stones (arrowhead) and gallbladder wall thickening consistent with cholecystitis. **(b)** Coronal contrast-enhanced CT image (portal venous phase) demonstrates mild thickening and enhancement of the intrahepatic and extrahepatic bile duct walls (arrows), which were initially interpreted as inflammatory changes. **(c)** Coronal contrast-enhanced T1-weighted MR image (portal venous to delayed phase) shows ring-like enhancement of the thickened bile duct walls (arrows), suggestive of an infiltrative process. **(d)** Coronal T2-weighted MR image (non-contrast) shows bile duct wall thickening and peribiliary edema (Glissonian sheath edema, arrowheads) with mild intrahepatic bile duct dilation; the thickened walls appear as high signal with a “double-track” sign along the portal venous branches.

After admission to the gastroenterology department (November 24, 2025), MRI of the upper abdomen with MRCP was performed on November 29, 2025. The imaging revealed bile duct wall thickening and peribiliary edema (Glissonian sheath edema) involving both intrahepatic and extrahepatic bile ducts, with mild intrahepatic bile duct dilation. On T2-weighted imaging, the thickened bile duct walls showed high signal intensity, and the peribiliary edema appeared as ill-defined linear or patchy high signal along the portal venous branches, with a “double-track” sign. On contrast-enhanced T1-weighted imaging, the bile duct walls demonstrated moderate ring-like enhancement in the arterial phase, with persistent ring-like enhancement in the portal venous to delayed phases (delayed enhancement). On diffusion-weighted imaging (DWI), the bile duct walls showed high signal with corresponding low ADC values, indicating restricted diffusion. No definitive vascular encasement was identified; however, the main portal vein and right portal branch appeared mildly narrowed. No significant hilar lymphadenopathy was detected. No discrete hilar or parenchymal mass was identified. The radiologist’s initial impression was “cholangitis” and “inflammatory stricture” of the common bile duct, with no suspicion of malignant biliary infiltration. In retrospect, the peribiliary edema (Glissonian sheath edema) and the pattern of delayed enhancement with restricted diffusion were key indirect signs suggestive of tumor infiltration rather than primary inflammation. Review of serial imaging showed progressive peribiliary involvement: from no detectable lesion in 2023, to subtle Glissonian sheath soft tissue on CT in October 2025, and finally to overt Glissonian sheath edema and bile duct dilation on MRCP in November 2025 (**Figures 1c, d**).

### 2.4 Diagnostic evaluation

The differential diagnosis evolved progressively during the patient’s hospital course. At admission, the patient’s symptoms and cholestatic laboratory profile raised several possibilities: biliary stones (given the known gallstones on CT), drug-induced liver injury (given recent chemotherapy), autoimmune/IgG4-related cholangitis, and malignant biliary obstruction (given known advanced ovarian cancer).

Drug-induced liver injury (DILI) was considered early, as the patient had recently received chemotherapy (albumin-bound paclitaxel, carboplatin, and bevacizumab) in October 2025. In the first few days, hepatoprotective therapy and antibiotics were initiated. Autoimmune and IgG4-related cholangitis were quickly excluded by negative serology (ANA, autoimmune hepatitis panel, IgG4, hepatitis A–E, liver fluke). However, after several days of treatment, a dissociation was observed: transaminases decreased while bilirubin progressively worsened (**Supplementary Table 1**), suggesting mechanical obstruction rather than hepatocellular injury. We then performed abdominal MRI with MRCP, primarily to assess disease progression (given the patient’s known ovarian cancer) and to determine whether a gallstone had migrated into the common bile duct. MRCP did not show evidence of bile duct stones, but it did reveal diffuse bile duct wall thickening, peribiliary edema, multifocal strictures, and mild intrahepatic dilation. These findings raised suspicion for a biliary process but remained inconclusive regarding etiology.

To further investigate, we proceeded with ERCP on day 11 (December 5, 2025) (**Figure 2a**). Cholangiography demonstrated a patent common bile duct without complete obstruction. However, a long-segment stricture was identified in the upper common bile duct (**Figures 2a**, arrow), with poor opacification of the affected segment. However, under cholangioscopic (SpyGlass) visualization, the mucosa of the entire common bile duct and intrahepatic bile ducts appeared diffusely rough, edematous, and rigid (**Figure 2b**)—findings consistent with a sclerosing cholangitis pattern. At this intraprocedural stage, we considered both primary sclerosing cholangitis (PSC) and secondary sclerosing cholangitis (SSC). PSC was deemed unlikely because it requires the absence of an identifiable secondary cause, whereas our patient had known advanced ovarian cancer; PSC is also strongly associated with inflammatory bowel disease, which this patient did not have. SSC due to malignant infiltration was considered the more likely etiology. No single dominant short-segment stricture suitable for stenting was identified. A nasobiliary drainage tube was placed for temporary drainage; however, less than three hours after the procedure, the patient requested removal of the tube because of intolerable discomfort. Thus, the primary diagnostic value of ERCP was to obtain tissue for histological confirmation. Forceps biopsy was obtained from the most abnormal-appearing mucosa in the upper common bile duct under direct cholangioscopic visualization; brush cytology was not performed. Although the exact number of tissue fragments was not recorded, the aggregate specimen size was approximately 0.1 cm. The pathology laboratory performed deeper sectioning of the specimen. Histopathology revealed fibrous tissue containing several small micropapillary clusters of atypical cells within the stroma, consistent with carcinoma. The limited sample size precluded extensive molecular profiling, and we acknowledge that the biopsied material was scant.

**Figure 2:**
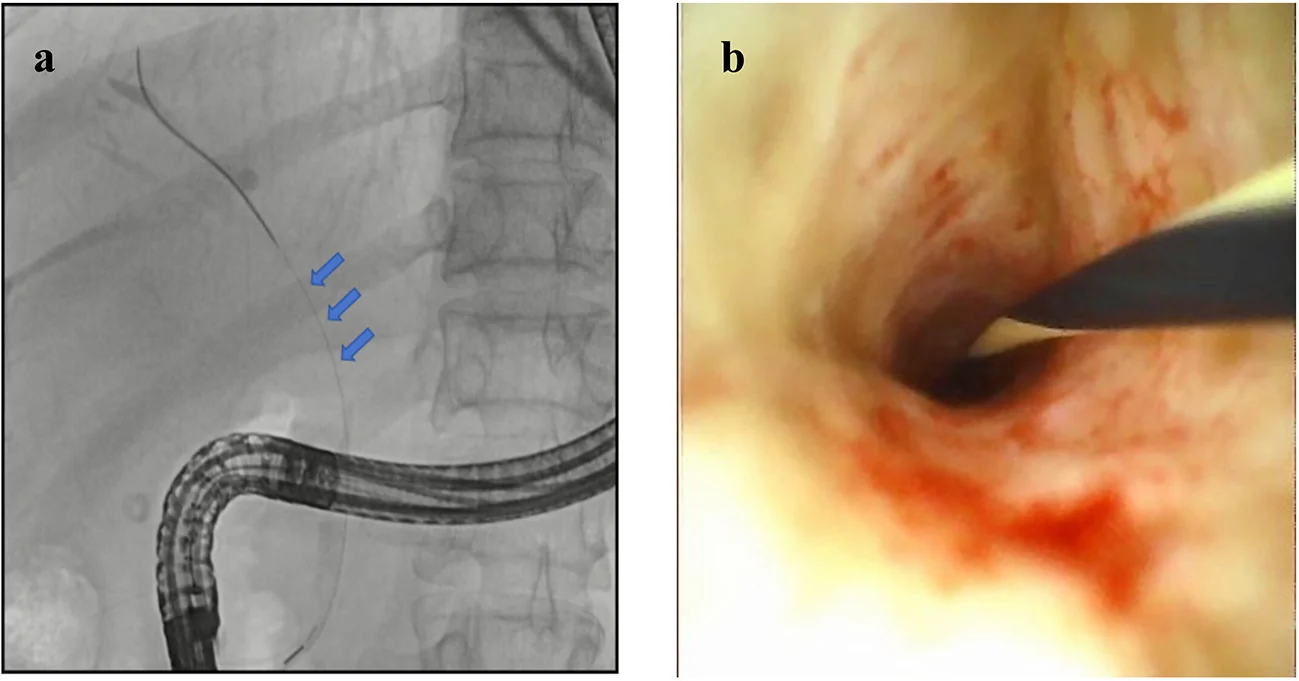
**(a)** ERCP showing a long-segment stricture in the upper common bile duct with poor opacification (arrow). **(b)** Cholangioscopic (SpyGlass) view showing diffusely rough, edematous, and rigid mucosa of the common bile duct.

Immunohistochemistry was performed using the following antibodies (clone, manufacturer, and positivity criteria): CK7 (clone MX053, Fuzhou Maixin Biotech Co., Ltd.; cytoplasmic positivity), CK19 (clone MX054, Fuzhou Maixin Biotech Co., Ltd.; cytoplasmic positivity), PAX-8 (clone MX062, Fuzhou Maixin Biotech Co., Ltd.; nuclear positivity), P16 (clone MX007, Fuzhou Maixin Biotech Co., Ltd.; diffuse cytoplasmic and nuclear positivity), p53 (clone MX008, Fuzhou Maixin Biotech Co., Ltd.; nuclear positivity, diffuse strong staining in >50% of tumor cells, interpreted as overexpression, consistent with mutant-type TP53), CEA (clone MX068, Fuzhou Maixin Biotech Co., Ltd.; cytoplasmic positivity, focal), ER (clone SP1, Fuzhou Maixin Biotech Co., Ltd.; nuclear positivity), and Ki-67 (clone MAX006, Fuzhou Maixin Biotech Co., Ltd.; nuclear positivity). The immunophenotype was: CK7+, CK19+, PAX-8+, P16+, p53 (overexpression)+, CEA (focal)+, ER−, Ki-67 high (**Figures 3a–d**), confirming metastatic high-grade serous carcinoma of ovarian origin involving the common bile duct wall. We acknowledge that this biopsy was obtained from the extrahepatic bile duct and did not sample the intrahepatic Glissonian sheath; the interpretation of diffuse sheath infiltration is based on radiological-pathological correlation.

**Figure 3:**
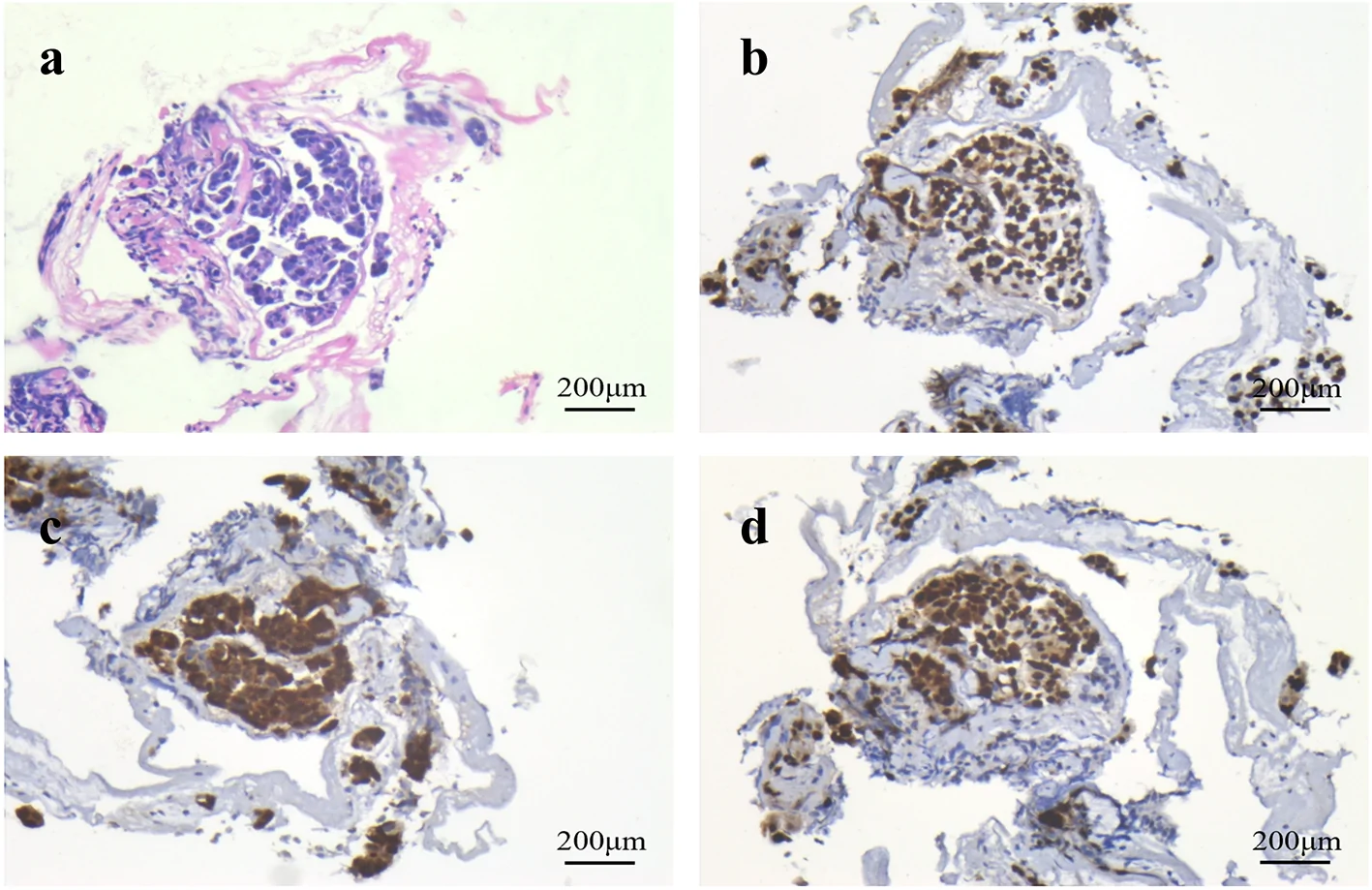
**(a)** Hematoxylin and eosin (HE) stain showing micropapillary clusters of atypical cells. **(b)** PAX-8 nuclear positivity. **(c)** P16 cytoplasmic and nuclear positivity. **(d)** Mutant-type p53 diffuse strong nuclear positivity. Scale bar for all panels=200μm. Original magnification:×100.

We attempted to retrieve the archival slides from the patient’s original ovarian tumor resection (April 2022) for direct comparison, but they were no longer available because the original surgery was performed at another hospital and the slides were not transferred to our institution. However, the original pathological reports from that external hospital were available. The initial report (April 2022) diagnosed high-grade serous carcinoma with the following immunophenotype: PAX-8 (+), WT-1 (+), p53 (diffuse strong +), CK7 (partial +), ER (partial weak +), and Ki-67 (approximately 80%). The second report (June 2022), obtained after neoadjuvant chemotherapy, confirmed the same diagnosis in the ovaries with treatment-related changes (degeneration and necrosis), while the resected specimens from the liver, spleen, and diaphragm showed no viable tumor cells, indicating a pathologic complete response (pCR). The biliary biopsy showed a concordant immunophenotype: PAX-8 (+), p53 (overexpression)+, CK7 (+), ER (−), and Ki-67 high. The shared PAX-8 positivity and p53 overexpression pattern strongly support a common origin, confirming that the biliary lesion represents metastatic ovarian carcinoma rather than a primary biliary malignancy. The slight discordance in ER expression (weak positive in the primary tumor versus negative in the metastasis) is not unexpected, as hormone receptor status may change during tumor progression and after chemotherapy.

### 2.5 Therapeutic intervention and outcome

After the pathological diagnosis was confirmed, a multidisciplinary team meeting (radiology, pathology, oncology, surgery, gastroenterology) was convened. The team concluded that surgical resection was contraindicated due to extensive metastases (peritoneal, retroperitoneal/mediastinal lymph nodes, vertebral). The oncology team noted that although alternative systemic therapy options remained available, the patient had experienced significant chemotherapy-related toxicity with insufficient treatment cycles, and her disease had progressed despite prior chemotherapy and targeted therapy. More importantly, further systemic treatment was precluded by progressive hyperbilirubinemia and hepatic dysfunction. The oncology team recommended deferring additional chemotherapy or targeted therapy until liver function improved.

Regarding biliary drainage, the team revisited the question in light of the pathological diagnosis. The following specific considerations informed the decision against additional drainage procedures (1): the ERCP findings had shown a patent lumen with diffuse mucosal abnormalities rather than a focal stricture (2); no single dominant short-segment lesion suitable for stenting had been identified (3); the patient had already demonstrated intolerance to the nasobiliary drain, requesting its removal within three hours due to severe discomfort; and (4) the obstructive process was attributable to diffuse tumor infiltration of the biliary tree rather than a focal obstruction. During the same multidisciplinary discussion, palliative local radiotherapy using a radioactive particle stent was considered as a theoretical option. However, the team specifically noted that radiation-induced edema might temporarily exacerbate biliary obstruction in this setting, and given the diffuse nature of the infiltrative process, no clear benefit could be assured. This option was therefore not pursued.

Given the diffuse nature of the biliary involvement, the absence of a targetable lesion, the patient’s prior intolerance to drainage, and her expressed preference for supportive care over further invasive interventions in the setting of advanced metastatic disease, the team concluded that additional endoscopic or percutaneous drainage procedures were unlikely to provide meaningful clinical benefit. The risks and uncertain benefit of biliary stenting in the setting of diffuse sclerosing cholangitis-like changes are well recognized, including the potential for stent-induced stone formation and procedure-related complications (6–8). The patient received supportive care with hepatoprotective agents (diammonium glycyrrhizinate) and ursodeoxycholic acid. These agents were used as supportive measures, not as definitive treatment for the malignant obstruction. The central therapeutic challenge remained the inability to achieve durable biliary drainage in the setting of diffuse tumor infiltration, compounded by progressive hepatic dysfunction that precluded continuation of effective systemic chemotherapy or targeted therapy. She was discharged against medical advice on December 12, 2025. One month after discharge, she continued to attend outpatient clinic visits and remained in contact with our team; her jaundice persisted, and she reported persistent fatigue, anorexia, and poor sleep. Subsequent telephone follow-up approximately six months after discharge revealed that the patient had died; the exact date of death was not confirmed, as further details were not pursued out of respect for the family’s feelings. The overall clinical course and timeline are summarized in **Figure 4**.

**Figure 4:**
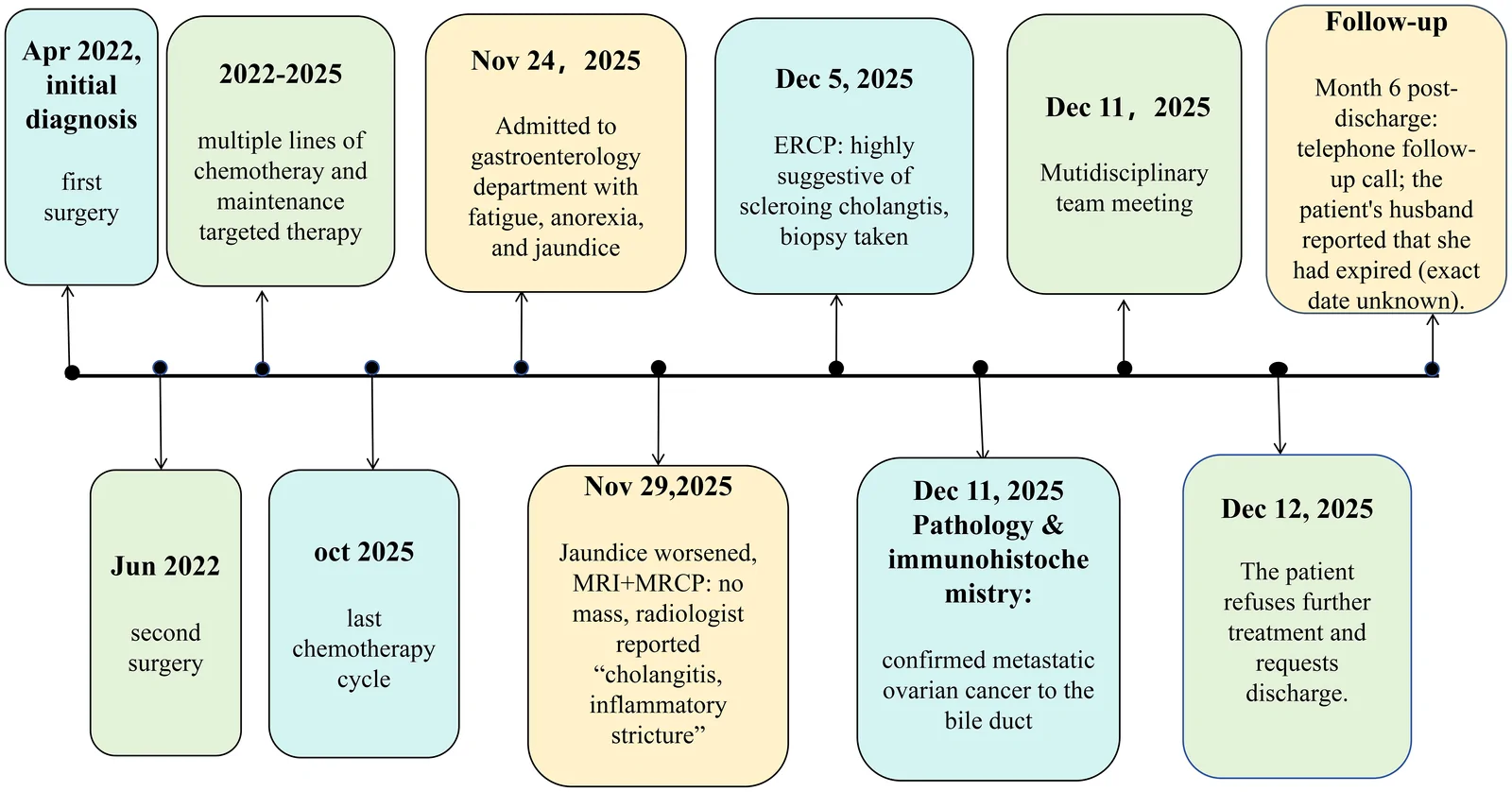
Timeline of the patient’s clinical course, diagnostic workup, and outcomes. Detailed treatment chronology is described in the Case Description, “Patient information and past medical history” section.

## 3 Discussion

Bile duct metastasis from ovarian cancer is extremely rare, typically described as discrete hilar masses causing obstructive jaundice. Shijo et al. reported a 10-mm solid hilar tumor confirmed as metastatic ovarian cancer after resection (4). Puvvada et al. described caudate lobe metastasis causing extrinsic compression of the common bile duct (5). In both reports, metastatic lesions presented as identifiable masses. In contrast, the present case demonstrates a pattern in which the clinically relevant biliary component did not present as a discrete mass on CT or MRCP, but rather as diffuse infiltrative involvement of the Glissonian sheath, radiologically mimicking sclerosing cholangitis (9). This observation suggests that biliary metastasis from ovarian cancer may not always present as an identifiable mass, and underscores the importance of tissue diagnosis when cross-sectional imaging shows biliary abnormalities without a discrete lesion.

### 3.1 Pathophysiological basis of Glissonian sheath infiltration

We acknowledge that the pathological evidence of metastasis was obtained from the extrahepatic common bile duct, which is anatomically distinct from the intrahepatic Glissonian sheath. The imaging findings, however, suggested intrahepatic peribiliary involvement. With this distinction in mind, the anatomical basis for the radiological pattern lies in the Glissonian sheath—a connective tissue envelope that surrounds the portal triad (portal vein, hepatic artery, and bile duct) within the liver (10). This sheath is continuous with the subperitoneal space and contains lymphatic vessels, which may serve as a conduit for tumor spread (11). In this case, ovarian cancer cells did not form an expansile mass; instead, we hypothesize that ovarian cancer cells may have spread along the peribiliary lymphatic spaces in a diffuse, infiltrative pattern. If tumor cells indeed progressively involved the periductal connective tissue, this could explain why the bile duct wall became thickened and non-compliant—functionally obstructed even without a luminal mass. However, we acknowledge that this mechanism is inferred from imaging-pathology correlation and was not directly observed histologically (12). The peribiliary edema seen on MRCP may represent a combination of tumor infiltration and secondary inflammatory changes, rather than pure inflammation alone. In retrospect, after pathological confirmation of malignancy, the progressive peribiliary abnormalities were considered suggestive of infiltrative metastatic disease, with possible associated inflammatory or edematous changes. We acknowledge that infection or inflammation may coexist with malignant obstruction, and that the imaging findings initially interpreted as cholangitis/cholecystitis may have reflected a mixed process rather than a purely inflammatory or purely malignant condition. This pathophysiological mechanism explains why the patient developed progressive obstructive jaundice without any radiologically detectable mass. Notably, the phenomenon of diffuse peribiliary infiltration without a mass lesion is not unique to ovarian cancer, a similar pattern of periductal infiltration along Glisson’s sheath via lymphatic vessels has been well documented in intrahepatic cholangiocarcinoma (13). Hoerder et al. also recognized that disseminated intrahepatic infiltration can cause biliary obstruction without a visible mass (14), and our case adds to the limited literature on metastatic ovarian cancer presenting with this pattern. The distinction between primary and secondary sclerosing cholangitis (SSC) is particularly relevant to this case. SSC is morphologically similar to PSC but originates from a known pathological process, and the diagnosis of PSC requires exclusion of secondary causes (15). SSC has been reported in association with various malignancies, including lymphoma, prostate cancer, gallbladder cancer, and ovarian cancer (16). Lew et al. reported a case of SSC due to lymphatic spread from ovarian cancer, in which ERCP revealed irregular hepatic ducts consistent with sclerosing cholangitis, but no malignant cells were identified on biliary sampling; the diagnosis was ultimately established by liver biopsy (17). This case shares important similarities with our patient, including the presence of diffuse biliary strictures and the diagnostic challenge of distinguishing malignant infiltration from sclerosing cholangitis. SSC can result from infectious, drug-induced, ischemic, obstructive, and autoimmune etiologies (17); in our patient, the underlying ovarian cancer provided a clear etiology for SSC. These examples support the clinical relevance of our observation, demonstrating that infiltrative peribiliary spread without a detectable mass is a “known phenomenon” across different tumor types, though it remains uncommon. Several potential routes of metastatic dissemination could explain the peribiliary involvement in this patient. Lymphatic extension along the peribiliary plexus is one plausible mechanism. However, hematogenous dissemination to the bile duct wall, microscopic peritoneal seeding along the hepatoduodenal ligament, or direct extension from hilar lymph node metastases could also contribute, either alone or in combination. In this patient, no discrete hilar lymphadenopathy was identified on imaging, but microscopic nodal involvement cannot be excluded. The exact route of spread remains uncertain, and multiple mechanisms may have coexisted.

### 3.2 Why did cross-sectional imaging fail to detect the metastasis?

The cancer cells infiltrated the loose connective tissue within the Glissonian sheath in a diffuse, infiltrative pattern rather than forming an expansile mass (18). This peribiliary lymphatic infiltration does not create a sharp interface with surrounding parenchyma and therefore does not appear as a typical contrast-enhancing nodule (19). Instead, it manifests as subtle bile duct wall thickening, peribiliary edema, and a benign-appearing stricture—easily misinterpreted as cholangitis. Indeed, a similar phenomenon has been described in other malignancies. Matsumoto et al. reported a case of metastatic lung adenocarcinoma in which tumor cells infiltrated multiple bile ducts without forming a mass, leading to imaging findings indistinguishable from sclerosing cholangitis (20). The existence of such cases across different primaries suggests a shared biological behavior—tumor tropism for the peribiliary lymphatic plexus—that warrants further investigation.

### 3.3 Diagnostic and therapeutic implications

This case carries several important clinical lessons. From a diagnostic standpoint, it reinforces the critical role of ERCP with biopsy in cancer patients with unexplained cholestasis, even when cross-sectional imaging shows no mass (21). Despite the minute biopsy specimen (0.1 cm), histopathology revealed micropapillary clusters of atypical cells, and immunohistochemistry confirmed ovarian origin (PAX-8+, CK7+, p53 overexpression). PAX-8 is a highly useful marker of Müllerian origin in the appropriate clinical context, particularly when combined with morphology and the known history of ovarian carcinoma. In this case, PAX-8 positivity, together with the mutant-type p53 staining pattern and CK7+/ER− profile, strongly supported ovarian origin and helped distinguish metastatic ovarian cancer from primary cholangiocarcinoma (which is typically PAX-8 negative) (22). The p53 overexpression pattern in the biliary biopsy was consistent with that of the primary ovarian tumor, further supporting the diagnosis of metastatic ovarian carcinoma. WT-1 was positive in the primary ovarian tumor, but was not tested on the biliary biopsy due to limited tissue availability. The serial CA-125 measurements in this case also provide a valuable diagnostic lesson. CA-125 remained within normal limits during olaparib maintenance therapy in early 2023, rose sharply with disease progression in March 2024, decreased after chemotherapy in April 2025, but subsequently rose again with new metastatic spread in September 2025. However, an apparent discordance was noted: the patient had a partial radiographic response (lymph node shrinkage) in April 2025, yet CA-125 remained elevated at 348.3 U/mL. This phenomenon may be explained by a mixed response, where some lesions responded to treatment while others progressed (23). In ovarian cancer, mixed responses are increasingly recognized (24). This case highlights the importance of interpreting CA-125 trends in conjunction with imaging findings, and recognizing that discordance may indicate mixed response or disease progression not yet apparent on imaging. This case also illustrates the clinical challenge of indeterminate biliary strictures—strictures that evade definitive diagnosis after standard evaluation including cross-sectional imaging and ERCP-based tissue sampling (25). While the majority of indeterminate biliary strictures are malignant, up to one-third are benign. In patients with a known malignancy, however, progressive segmental or diffuse bile duct wall thickening, unexplained periportal soft tissue changes, and cholestasis—even without a discrete mass—should prompt active pursuit of tissue diagnosis. In our case, the patient’s known ovarian cancer history raised suspicion for malignant infiltration, but the ERCP findings initially provided a direction for differential diagnosis—a sclerosing cholangitis-like pattern—and the correct diagnosis of metastatic ovarian carcinoma was ultimately established only through targeted biopsy and immunohistochemistry. From a therapeutic standpoint, our case illustrates the dilemma once such infiltrative peribiliary metastasis occurs. Effective biliary drainage could not be established. The underlying nature of the stricture—diffuse, rigid encasement of the bile duct by tumor rather than a discrete, short-segment obstruction—made biliary stenting unlikely to be effective even if attempted. Surgical resection was precluded by widespread metastases, and systemic therapy was limited by progressive liver dysfunction and hyperbilirubinemia. These challenges highlight the aggressive nature of this rare metastatic pattern and underscore the importance of early recognition and multidisciplinary management. Finally, rare metastatic phenotypes such as the one described here may be better understood through transcriptomic approaches rather than morphology alone. Network-based methods such as weighted gene co-expression network analysis (WGCNA) can identify coordinated biological programs associated with dissemination and unusual organ tropism, and may help generate hypotheses about peribiliary lymphatic spread (26). However, no transcriptomic analysis was performed in this patient, and this remains a hypothesis-generating perspective for future research.

## 4 Limitations

This report has several limitations. First, although the patient died after discharge according to family report, the exact cause of death and precise survival time could not be verified from medical records because no autopsy was performed and the patient did not die in our hospital. The family was unwilling to provide further details at the time of follow-up, and out of respect for their feelings, we did not pursue additional inquiries. Second, effective biliary drainage could not be achieved because the patient was unable to tolerate the nasobiliary drain; therefore, the potential benefit of drainage in relieving jaundice could not be fully assessed. Whether the patient would have responded to sustained drainage or stenting remains unknown. Third, although the diagnosis of metastatic ovarian cancer was firmly established by immunohistochemistry, the small biopsy size (0.1 cm) precluded further molecular profiling. Fourth, the archival slides of the primary ovarian tumor were not available for direct morphological comparison because the original surgery was performed at another hospital and the slides were not transferred to our institution. Molecular testing of the primary tumor was not performed at the time of diagnosis. Additionally, surgical margin status and hilar lymph node involvement were not specifically evaluated in the pathology reports from the second surgery, as these details were not documented in the original records. Furthermore, no autopsy was performed, and no large tissue specimen from the affected hepatic portal tracts was obtained; therefore, the full histological extent of the biliary disease and the anatomical localization of the Glissonian sheath abnormalities could not be confirmed. Whether the radiological changes throughout the biliary tree corresponded to metastatic carcinoma remains uncertain. Similarly, the possibility of diffuse microscopic hepatic parenchymal infiltration cannot be excluded, as no diffuse parenchymal metastases were visible on imaging, but the absence of visible lesions does not entirely rule out microscopic involvement. This limitation is relevant when considering why biliary drainage, even if achieved, might not have fully reversed the liver dysfunction. Nevertheless, the combination of progressive periportal and peribiliary imaging abnormalities, malignant cells identified in the biliary biopsy, and the clinical context strongly supported the diagnosis of metastatic biliary involvement. The diagnostic value of this case-–demonstrating an extremely rare pattern of Glissonian sheath infiltration mimicking sclerosing cholangitis-–remains robust.

## 5 Conclusion

This case demonstrates that metastatic high-grade serous ovarian carcinoma can involve the biliary tract and may produce diffuse infiltrative radiological and cholangiographic abnormalities without a conventional mass, mimicking sclerosing cholangitis. However, this case does not establish that all radiological abnormalities represent histologically proven Glissonian sheath infiltration, nor does it confirm peribiliary lymphatic spread as the specific route of dissemination. Clinicians should maintain a high index of suspicion for biliary metastasis in cancer patients with unexplained cholestasis, even when cross-sectional imaging is unrevealing. In cancer patients with unexplained cholestasis and biliary abnormalities, tissue diagnosis should be actively pursued. ERCP with targeted biliary biopsy was particularly valuable in this case because it allowed direct sampling of the affected bile duct while demonstrating the cholangiographic appearance. However, other approaches—such as EUS-guided sampling, percutaneous biopsy, or cholangioscopy-guided biopsy—may be appropriate depending on the anatomy and clinical context. The key message is that malignancy should be considered even when cross-sectional imaging shows no discrete mass.

## 6 Patient perspective

The patient had been battling ovarian cancer for over three years, with multiple recurrences and lines of chemotherapy. She experienced cycles of hope with each new treatment, only to face subsequent disease progression. When she developed jaundice, she initially attributed it to treatment-related effects rather than disease progression, partly because she had recently undergone a CT scan during her oncology admission that had shown decreased lymph node size—so she did not suspect a major problem. At the time of admission, her bilirubin levels were only mildly elevated, and she had not even noticed that she was jaundiced. However, as her jaundice progressively worsened during hospitalization, she began to suspect that her tumor might have progressed further. She became increasingly anxious. She had already endured years of aggressive treatment and its side effects, and the prospect of further interventions—especially with uncertain benefit—weighed heavily on her. She actively participated in treatment decisions and, after understanding that further curative treatment was not feasible, prioritized her quality of life and time with family. She discussed her situation with her family, who supported her decision to be discharged against medical advice to spend her remaining time at home. She continued to attend outpatient follow-up one month after discharge, but her symptoms persisted. Approximately six months after discharge, during a telephone call with her husband, the family reported that she had died; the exact date was not confirmed. Written informed consent for publication was obtained from the patient during hospitalization.

## Data Availability

The original contributions presented in the study are included in the article/**Supplementary Material**. Further inquiries can be directed to the corresponding author.

## Supplementary material

Supplementary Figure 1 Serial CA-125 levels during the disease course:Arrows indicate key clinical events: pCR after neoadjuvant chemotherapy (June 2022); disease progression with retroperitoneal lymphadenopathy and bone metastases (March 2024); partial response to chemotherapy (April 2025); new metastases in mesentery, peritoneum, and mediastinal lymph nodes (September 2025); and admission to our department (November 2025). The trend shows a clear correlation between CA-125 elevation and clinical/radiographic progression.
